# Investigating drug repositioning opportunities in FDA drug labels through topic modeling

**DOI:** 10.1186/1471-2105-13-S15-S6

**Published:** 2012-09-11

**Authors:** Halil Bisgin, Zhichao Liu, Reagan Kelly, Hong Fang, Xiaowei Xu, Weida Tong

**Affiliations:** 1 Department of Information Science, University of Arkansas at Little Rock, 2801 S. University Ave., Little Rock, AR 72204-1099, USA; 2 Division of Bioinformatics and Biostatistics, National Center for Toxicological Research, US Food and Drug Administration, 3900 NCTR Road, Jefferson, AR 72079, USA; 3 ICF International Company at FDA's National Center for Toxicological Research, 3900 NCTR Rd, Jefferson, AR 72079, USA

## Abstract

**Background:**

Drug repositioning offers an opportunity to revitalize the slowing drug discovery pipeline by finding new uses for currently existing drugs. Our hypothesis is that drugs sharing similar side effect profiles are likely to be effective for the same disease, and thus repositioning opportunities can be identified by finding drug pairs with similar side effects documented in U.S. Food and Drug Administration (FDA) approved drug labels. The safety information in the drug labels is usually obtained in the clinical trial and augmented with the observations in the post-market use of the drug. Therefore, our drug repositioning approach can take the advantage of more comprehensive safety information comparing with conventional de novo approach.

**Method:**

A probabilistic topic model was constructed based on the terms in the Medical Dictionary for Regulatory Activities (MedDRA) that appeared in the Boxed Warning, Warnings and Precautions, and Adverse Reactions sections of the labels of 870 drugs. Fifty-two unique topics, each containing a set of terms, were identified by using topic modeling. The resulting probabilistic topic associations were used to measure the distance (similarity) between drugs. The success of the proposed model was evaluated by comparing a drug and its nearest neighbor (i.e., a drug pair) for common indications found in the Indications and Usage Section of the drug labels.

**Results:**

Given a drug with more than three indications, the model yielded a 75% recall, meaning 75% of drug pairs shared one or more common indications. This is significantly higher than the 22% recall rate achieved by random selection. Additionally, the recall rate grows rapidly as the number of drug indications increases and reaches 84% for drugs with 11 indications. The analysis also demonstrated that 65 drugs with a Boxed Warning, which indicates significant risk of serious and possibly life-threatening adverse effects, might be replaced with safer alternatives that do not have a Boxed Warning. In addition, we identified two therapeutic groups of drugs (Musculo-skeletal system and Anti-infective for systemic use) where over 80% of the drugs have a potential replacement with high significance.

**Conclusion:**

Topic modeling can be a powerful tool for the identification of repositioning opportunities by examining the adverse event terms in FDA approved drug labels. The proposed framework not only suggests drugs that can be repurposed, but also provides insight into the safety of repositioned drugs.

## Background

Drug repositioning (or repurposing) refers to the action of discovering new uses or indications for the existing drugs. Pharmaceutical companies, academic researchers, and government agencies have focused resources on repositioning as a way to augment the slowing drug discovery pipeline due to shorter development timelines and lower risk concerns compared to new drug development [1,2]. Traditionally, drug repositioning mainly relied on serendipity or 'happy accidents'; the classic examples are Viagra (sildenafil) and Thalomid (thalidomide) [3]. *In silico *approaches that provide a systematic way to explore drug repositioning opportunities have gained acceptance [4,5].

*In silico *drug repositioning seeks opportunities based on retrieving and organizing different data profiles. One rich repositioning resource is the NCGC Pharmaceutical Collection (NPC), which contains all approved small-molecule drugs and can be surveyed using ultra high-throughput screening assays to systematically explore repositioning opportunities across human diseases, particularly rare and neglected ones [6]. Kinnings et al. [7] applied a support vector machine (SVM) approach using molecular docking scores based on protein structure data from Protein Data Bank (PDB) and identified a phosphodiesterase inhibitor, Comtan, that could be potentially repurposed to target *Mycobacterium tuberculosis*. Dudley et al. [8] discovered the anticonvulsant topiramate's application to inflammatory bowel disease (IBD) by analyzing gene expression data from NCBI's Gene Expression Omnibus (GEO) on IBD samples and 164 small-molecule drug compounds. Electronic medical records and PubMed are also used for *in silico *drug repositioning via text mining [9,10].

There are several conceptual approaches to *in silico *drug repositioning, which mainly focus on how similarity between the drug space and disease space is assessed and quantified [11-13]. Phenotypic data such as side effects are an informative source of similarity assessment and has been used in drug repurposing. Campillos et al. [14] investigated off-target effects by integrating side effect profiles with chemical structures and identified several new drug-target interactions. They validated 13 implied drug-target relations by *in vitro *binding assays, of which 11 revealed inhibition constants equal to or less than 10 mM. Lun et al. [15] detected 3175 side effect and disease relationships and applied an *in silico *method to predict repositioning opportunities. Brouwers et al. [16] applied a network approach to compare the relationship between side effect similarity and off-targets shared by drugs.

There are several sources to obtain the side effect data, but this effort focused primarily on U.S Food and Drug Administration (FDA) approved labels for marketed drugs. The main reason for this preference is that a label description is based on the observations in both clinical trials and post-marketing surveillance, and so it represents a more systematic and comprehensive information resource than what is available from sporadic adverse event reporting after a drug is marketed. This research focuses on three related sections of the drug label, Boxed Warning (BW), Warnings and Precautions (WP), and Adverse Reactions (AR), in order to establish a robust relationship between drugs and side effects, rather than a broader, less focused data source such as the Side Effect Resource (SIDER) [17].

Drug labels require text mining techniques to extract useful information. Mapping documents to a lower dimensional concept space for semantic analysis is a well studied subject in information retrieval and text mining [18]. Recently, topic modeling based on the graphical model Latent Dirichlet Allocation (LDA) [19] has been applied to biological research [20,21]. Topic modeling was applied to the discovery of "topics" from textual drug labels, where a topic is a set of words that represents a specific concept. Our previous work in this field focused on whether topic modeling could cluster drugs into biologically meaningful groups from either a safety or therapeutic perspective. The topic models we developed successfully grouped drugs with similar safety concerns [22].

There are several in silico ways to detect drug repositioning opportunities. Among them, similarity based approaches have been proposed and have several successful examples [14]. Similarity measure can be based on chemical space, genomic space and clinical knowledge space. Here, we employed the side effect data and used topic modeling to search for repositioning opportunities.

**Figure 1:**
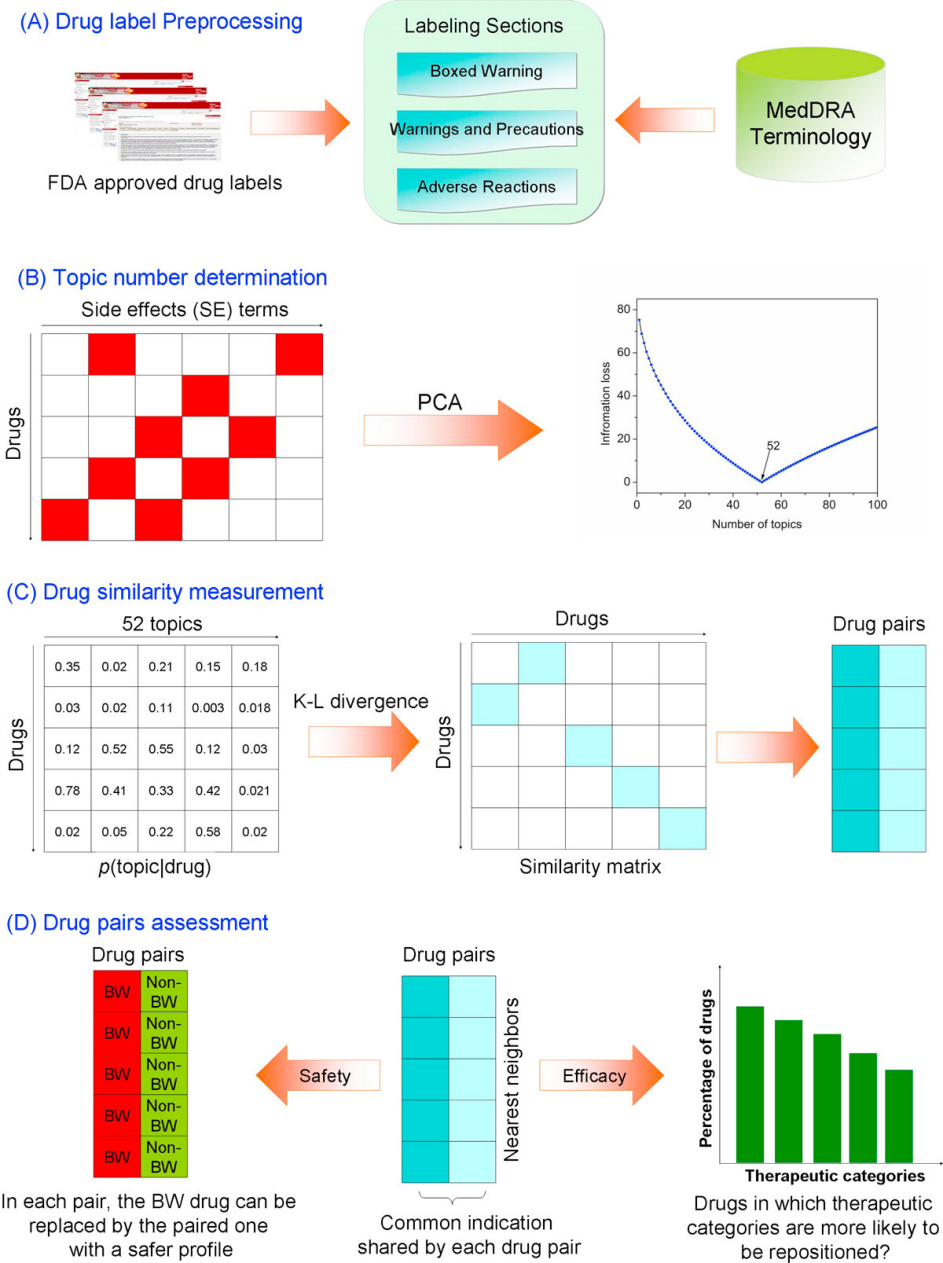
**Flowchart of the study**.

In this study, we hypothesized that drugs with similar side effect profiles likely share the same indications. In contrast to our previous study [22], we wanted to discover the semantic relationship between drugs. We then used this relationship as a measure of similarity between drug pairs and used that similarity to identify potential repositioning opportunities. A flowchart of the topic modeling approach we used is shown in Figure 1. Our results suggest that this approach could find alternative drugs for a particular indication. Furthermore, safer alternatives could also be identified using this approach to potentially replace BW drugs. We also identified several therapeutic categories that were over-represented with repositioning candidates, indicating that drugs in these therapeutic categories may be more likely to be repositioning candidates.

## Materials and methods

### Drug label data set

DailyMed http://dailymed.nlm.nih.gov/dailymed/, a publicly available data source, lists FDA-approved labels of marketed drugs. Because a drug is often marketed with multiple brand names associated with multiple labels, we used the most recent label according to its effective date regardless of the brand name for each drug. Only drugs that are taken orally or by injection were examined in this study.

After identifying the drugs we would use, we parsed labels with XML formats. We used the three labeling sections related to the safety concerns (BW, WP, and AR) for further analysis. Information in these three sections contains not only safety concerns, but also adverse events and precautions that should be considered in the clinical use of the drug. We filtered raw text from the labels with standardized side effect (SE) terms in the Medical Dictionary for Regulatory Activities (MedDRA) http://www.meddramsso.com/ maintaining the lowest level terms consisting of 68,259 terms from 26 organs [23]. The SE profile contained 4,822 SE terms for each drug, which we used as the input matrix for topic modeling.

### Drug indication data set

One shortcoming of the drug labels is that the indication sections do not list the indications in a way that can be consistently matched with the terms in a database like MedDRA. In order to integrate pre-processed indication concepts, we utilized SIDER [17]http://sideeffects.embl.de/, which provides indications for 888 drugs. For each drug in both data sources, we integrated the side effect profile from the drug label and indication terms from SIDER. Integrating both sources resulted in 870 drugs.

### Topic modeling

A topic model is a statistical model of documents. A topic model or probabilistic latent semantic index (pLSI) is not a generative model, therefore it can not fully describe the dependency of documents, topics and words [24]. In a Latent Dirichlet Allocation model (LDA) a Dirichlet prior is introduced, so that not only the model is generative for new documents, but also the inference is more convenient [19]. The underlying concept of LDA is that a document has a mixture of topics and that each word is selected with a probability given one of the document topics. For each document d, θ(*d*) = *P*(*z*) stands for the multinomial distribution over topics. Let *P*(*w*|*z*) be the probability distribution over words w given topic z. Then, document d can be generated by following two steps for each word *w_i _*(where *i *is the index for *i*-th word of document d): first, a topic *j *is selected with a probability of *P*(*z_i _*= *j*) based on the probability distribution *P*(*z*); second, a word *w_i _*is picked out with a probability of *P*(*w_i_*|*z_i_*=*j*). Therefore, the generative process prescribes the following distribution of words in document d:

(1) $$P\left(w_{i}\right)=\overset{T}{\underset{j=1}{\sum }}P\left(w_{i}|z_{i}=j\right)P\left(z_{i}=j\right)$$

where *T *is the number of topics.

### Determining number of topics

Like other dimension reduction methods in the literature, topic modeling aims to remove redundancy in addition to finding topics in the documents. The number of topics to be searched for is usually determined empirically or by some heuristic approaches such as seen in recent studies [25,26]. On the other hand, topic modeling can be also seen as a matrix factorization method. In this work we suggest a different heuristic approach to determine the number of topics. We first used Principal Component Analysis (PCA) on the drug-term matrix to attain the eigenvalues and then minimized the information loss as follows:

(2) $$\underset{k}{\text{argmin}}∥\overset{n}{\underset{i=1}{\sum }}e_{i}-\lambda \overset{n}{\underset{i=k+1}{\sum }}e_{i}∥$$

where λ is a penalty, which regularizes the information loss. We found that an optimal result is often achieved when λ = 2 in our study. In this case, the number of topics *k *is determined as follows:

(3) $$\underset{k}{\text{argmin}}∥\overset{k}{\underset{i=1}{\sum }}e_{i}-\overset{n}{\underset{i=k+1}{\sum }}e_{i}∥$$

### Drug distance assessment

After obtaining the topics, one of the outputs of this model is the probability distribution of topics for a given drug, i.e., *P*(*z*|*d*), where *z *and *d *represent the random variables for topics and drugs respectively. This conditional probability is a signature of the drug, which is used to assess the drug similarities. Ding, et al. proposed a similar signature for genes based on a distribution of topics, which is determined by a straightforward counting [27].

We used the Kullback-Leibler (K-L) divergence [28], a measure of the difference between two probability distributions *P *and *Q*, to calculate similarities between drugs based on conditional probabilities *P*(*z*|*d*). K-L divergence is given by:

(4) $$D_{KL}\left(P||Q\right)=\overset{}{\underset{i}{\sum }}P\left(i\right)\text{ ln}\frac{P\left(i\right)}{Q\left(i\right)}$$

In contrast to many metric measures, K-L divergence is asymmetric. Therefore, as the pairwise distance between drug *A *and *B*, *D*(*A, B*), we computed the following to symmetrize the relation:

(5) $$D\left(A,B\right)=\frac{D_{KL}\left(A||B\right)+D_{KL}\left(B||A\right)}{2}$$

### Common indication search

Using the pairwise symmetrized K-L distance defined in equation (5), we identified the nearest neighbor for each drug in the dataset. We then examined any common indication between a drug and its nearest neighbor. In order to generate a null distribution for each drug in the dataset we randomly chose a second drug and noted any common indications. We performed this procedure 10,000 times and recorded the percentage of trials in which a common indication was successfully located.

## Results

As shown in Figure 1, the study involves four steps: (1) drug label preprocessing - three sections of drug labels, BW, WP, and AR, were used and SE terms were extracted using MedDRA terminology for each drug; (2) topic number determination - PCA with an information loss criterion was employed to determine the optimal number of topics; (3) drug similarity measurement - the drug-topic conditional probability matrix was obtained by using topic modeling, based on which the drug-drug similarity matrix was obtained by calculating K-L divergence; and (4) drug-pair assessment - drug pairs were assessed from both therapeutic and safety perspectives based on their known shared indications.

### Number of topics

**Figure 2:**
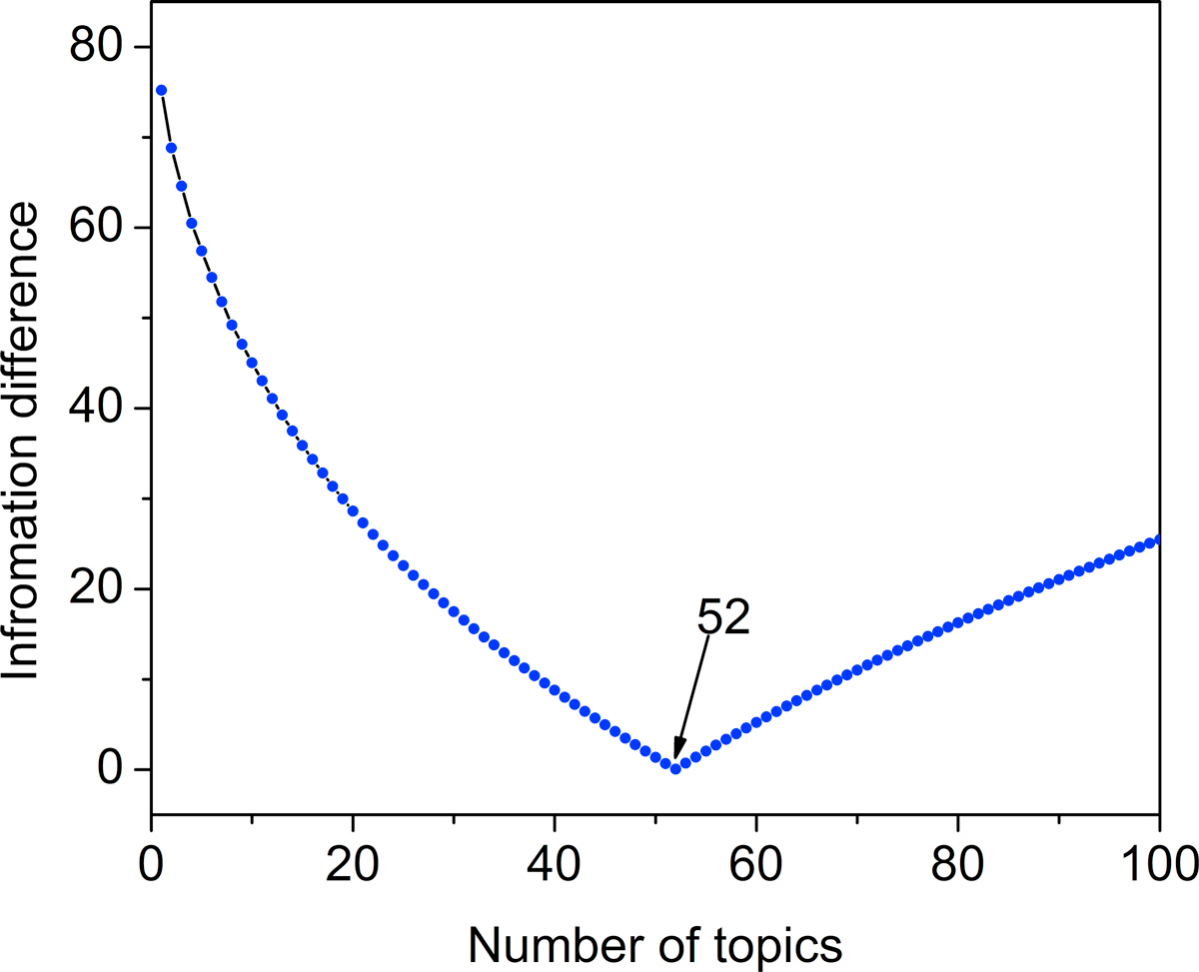
**Optimal number of topics: information loss is plotted vs. number of topics; the minimal information loss occurs when the number of topics equals 52**.

We obtained the number of topics using PCA with an information loss criterion as described in the Materials and Methods section. Figure 2 shows how the optimal number of topics was acquired via minimizing the information loss as described in Equation (3). The information loss reaches its minimum when the topic number is equal to 52. This means the original 4,822 SE profile can be represented by 52 topics.

### Drug pairs with common indication

**Figure 3:**
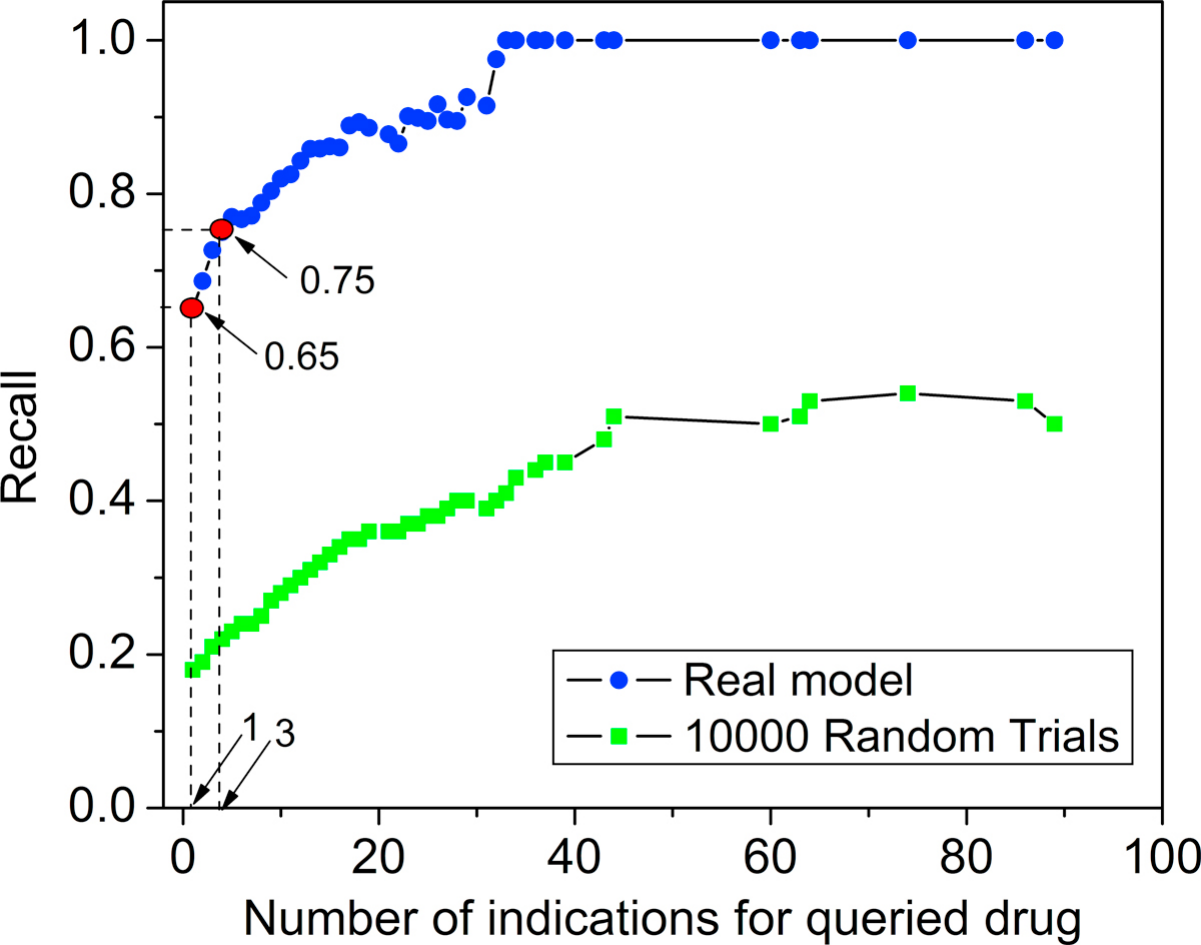
**Recall vs. number of indications: Drugs with a low number of indications have a lower chance to find a nearest drug with a common indication; The topic model consistently outperformed the random chance**. The red dots represent the recall of drugs with one and three indications, respectively.

After assessing the distance by symmetrized K-L divergence, we identified common indications for the closest drug pairs. Using this information we calculated recall, or the ratio of the number of pairs sharing an indication to the total number of pairs. The dotted blue line in Figure 3 shows how recall values increase as the number of indications for a given drug increases. Out of 870 closest drug pairs, 569 shared at least one common indication, which corresponded to a 65% recall. We expect that drugs with only one indication have a very low chance to share that indication with other drugs. Therefore, we recalculated the recall considering only those drug pairs where at least one drug had multiple indications. As shown in Figure 3, when the drugs in the query list have more than three indications, the recall reaches 75%, and grows rapidly as the number of indications increases.

We repeated the same procedure for the randomly selected drug pairs as a comparison (illustrated by the green dotted line in Figure 3). The result shows that both methods generated a similar trend, however, the real model consistently outperforms the random selection by a factor of 5.

### Safety issues in drug repositioning

Balancing safety and efficacy is a key goal in drug development. One of the aims of drug repositioning is to find safer drugs to replace currently prescribed drugs that may have safety concerns. A drug with a BW has been defined by the U.S. Code of Federal Regulations (21CFR201.57) to be capable of causing serious adverse reactions or even death [29,30]. If an alternative drug with fewer safety concerns can be identified, it would be a major benefit to public health. There are 342 drugs with a BW in this dataset. We examined the drugs paired with a BW drugs for each of the 342 BW drugs. We successfully identified potential safer alternatives (candidates without a BW) for 65 drugs, indicating that the proposed method may offer a new way to search safer drugs to replace ones with safety concern for the same indication. For instance, cefazolin is prescribed for urinary tract infections and has a BW, but our research suggests that a safer alternative, cefuroxime, may be used for the same disease.

### Drug repositioning opportunities for therapeutic categories

**Figure 4:**
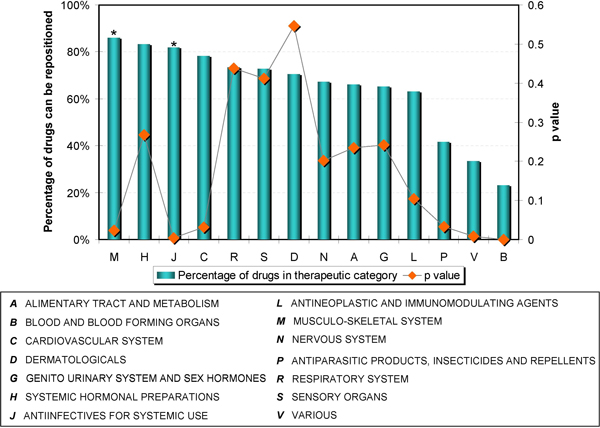
**The percentage of the drugs that might be repositioned and the corresponding *p *values for 14 therapeutic categories**. Two therapeutic groups (M = Musculo-skeletal system and J = Anti-infective for systemic use, labeled with asterisk) where over 80% of the drugs have a potential replacement.

We extracted the first level term from the Anatomical Therapeutic Chemical Classification System (ATC) http://www.who.int/classifications/atcddd/en/ for drugs involved in drug pairs that shared at least one common indication. Figure 4 shows the distribution of repositioning candidates identified by therapeutic category and the corresponding *p*-value for 14 therapeutic categories. For each therapeutic category, we calculated the percentage of drugs with a nearest neighbor sharing one or more common indications and used a Fisher's exact test to check if the observed distribution deviates significantly from the expected distribution.

Two therapeutic categories, M (Musculo-skeletal system) and J (Anti-infective for systemic use), had the highest percentage (86% and 82%, respectively) of drug pairs sharing common indications and statistically significant Fisher's Exact tests (*p*-values < 0.05). This suggests that drugs in both groups are more likely to be able to be repurposed. For example, most nonsteroidal anti-inflammatory drugs (NSAIDs), e.g., ibuprofen, belong to the Musculo-skeletal system category yet ibuprofen is a COX inhibitor and can initiate pain relief. The proposed method found that the nearest neighbor of indomethacin is ibuprofen. Indomethacin has an anti-Parkinson's effect [31], suggesting that ibuprofen might be effective for Parkinson's disease as well. Animal studies and clinical trials have demonstrated that ibuprofen can reduce the development of Parkinson's disease [32]. Since ibuprofen is an over-the-counter drug, the results demonstrate that our method has the ability to find safer alternative drugs for the treatment of the same disease.

## Discussion

Discovering new uses for an existing drug is challenging. Traditionally, repositioning opportunities were discovered mainly by chance or by expert opinion. An *in silico *approach to drug repositioning is an important contribution to the drug discovery pipeline by offering a comprehensive method for suggesting alternative therapeutic uses of existing marketed drugs.

In this work, we developed an *in silico *approach based on topic modeling. FDA approved drug labels were used because of their well-defined and well-structured terminology. In particular, we used topic modeling to calculate a probabilistic topic distribution of adverse event terms appearing in the sections related to safety issues for each drug. We then measured the distance between pairs of drugs by means of this probabilistic topic distribution. We considered a candidate for drug repositioning to be identified if the nearest neighboring drug shares a common indication. This method provides several notable advantages. First, with its unsupervised nature, topic modeling does not require *a priori *information about the drugs. Secondly, it offers clear and easily understandable criteria for determining if a drug pair contains a repositioning candidate. Lastly, even if a suggested drug pair does not share any common indications, it may be worth further investigation because one of them may have an unknown indication that could have potential application.

The advantage of topic modeling is that a document is linked to several topics and the relationship between documents is preserved via these topics. In this study, a drug is characterized by its label and the similarity between drugs is determined by the similarity in topics contained by the three sections of the labels dealing with side effects. For every drug label, the similarities captured by the topic distribution suggested a nearest neighbor. This implies that even when the content of the drug labels is not exactly the same, the topics may well be very close to each other. We compared the common indications of all closest drug pairs suggested by our model with that of random drug pairs. This analysis showed that the proposed method identified at least three times as many repositioning candidates than would be expected by chance alone The recall of this method was over 69%, while there is only a 19% chance that two randomly selected drugs will share a common indication. The difference not only demonstrates the potential success of the proposed approach, but also invites the investigation of the remaining 31% of drug pairs. For example, atomoxetine and theophylline do not appear to share a common indication given the information reported in the drug label. However, after searching the literature, we found that theophylline may also be a useful drug to treat Attention-Deficit/Hyperactivity Disorder (ADHD), as is atomoxetine [33]. Similarly imipramin and buproprion do not have any indications in common, but Jacobs et al. [34] reported that a trial of imipramin was undertaken and that it was found to be effective for smoking cessation, a new indication for buproprion, but not at a desirable level.

We also observed that some therapeutic groups appeared more frequently in the successful pairs. When those frequencies were normalized by the total frequencies for all pairs, certain ATC categories contained significantly more successful pairs than what would be expected by chance alone. This finding indicates that some therapeutic groups are more prone to have drugs with common indications, which implies that the chance of finding repositioning candidates among these drugs is high. Furthermore, the findings also suggest that drug repositioning opportunities might exist not only within the same category, but also among the higher-level groups as well.

While repositioning opportunities are being explored, safety issues cannot be neglected. In ideal circumstances, drugs with minimum risk and maximum efficacy should be the first choice for repositioning. In this regard, our approach draws attention to drug pairs that suggest a safer alternative for the same disease. The proposed approach offers a way of identifying a drug without a BW to substitute for a drug with a BW. As an example, auranofin is used to treat rheumatoid arthritis but has a BW. Our system identified a drug (meclofenamate, which does not have a BW) already known to be safer for this indication.

Drug efficacy and safety are among the most critical and challenging issues facing government agencies, pharmaceutical companies, and academic researchers. Since FDA-approved drug labels are the most comprehensive and reliable source for therapeutic and safety information about currently marketed drugs, they are critical for the development of a novel *in silico *drug repositioning method. As new drugs are approved, new labels are created. Additionally, after years of clinical use of drugs, updates to their drug labels may be made because knowledge about the drug may change. The dynamic nature of the drug labels also requires an appropriate text mining approach so that the temporal pattern in the drug labels can be utilized for a more powerful drug repositioning system. Although the current study only considered the most recent drug labels, drug labels can also be mined at varying time points by using a dynamic topic modeling approach. In addition to predicting repositioning opportunities, the dynamic approach may also enable the development of an alert system for pharmacovigilance purposes.

## Conclusions

This study investigated drug repositioning opportunities with an additional focus on safety analysis by performing topic modeling on FDA drug labels and measuring drug similarity by the number of discovered topics representing side effects. Our results demonstrated that drugs considered to be similar by this method may often be effective for the same disease. There are several benefits of this proposed approach: it may offer opportunities to reposition drugs without a BW to replace the drugs with BW; it may successfully identify therapeutic groups with the highest chance for drug repositioning, and the proposed method could offer a promising approach for pharmacovigilance.

## Disclaimer

The views presented in this article do not necessarily reflect those of the US Food and Drug Administration.

## Competing interests

The authors declare that they have no competing interests.

## Authors' contributions

HB and ZC, performed all calculations and data analysis, and wrote the first draft of manuscript. WT and XX developed the methods and had the original idea and guided the data analysis and presentation of results. HF and RK contributed to the data analysis, verified the calculations, and assisted with writing the manuscript. All authors read and approved the final manuscript.
